# Assessing reported cases of sexual and gender-based violence, causes and preventive strategies, in European asylum reception facilities

**DOI:** 10.1186/s12992-018-0365-6

**Published:** 2018-05-09

**Authors:** Charlotte Oliveira, Ines Keygnaert, Maria do Rosário Oliveira Martins, Sónia Dias

**Affiliations:** 1 Global Health and Tropical Medicine, GHTM, Instituto de Higiene e Medicina Tropical, IHMT, Universidade Nova de Lisboa, UNL, Rua da Junqueira 100, 1349-008 Lisbon, Portugal; 2 International Centre for Reproductive Health, Faculty of Medicine & Health Sciences, Ghent University, Ghent, Belgium

**Keywords:** Sexual and gender-based violence, Sexual violence, Refugees, Asylum-seekers, Migrants, Asylum reception facilities, Professionals, Causes, Prevention measures, Preventive strategy

## Abstract

**Background:**

Sexual and gender-based violence (SGBV) is a widespread public health problem and a violation of human rights rooted in gender and power inequities. Refugees, asylum-seekers and migrants living in European asylum reception facilities (EARF) are especially vulnerable to SGBV. To contribute to closing the gap on systematic and accurate evidence on SGBV, we aim to explore reported cases of SGBV, causes and preventable measures described by residents and professionals from EARF.

**Methods:**

We developed a cross-sectional study using the Senperforto project database. Semi-structured interviews were conducted with residents (refugees, asylum-seekers and unaccompanied minors) and professionals (service and health care providers) at EARF, in 7 European countries. We used IBM® SPSS software to analyze our data. Further, statistical tests – *Chi-square Test* and *Fisher’s exact test* (5% significance level) were conducted.

**Results:**

In total 562 respondents: 375 residents (R) and 187 professionals (P) participated in the study. The majority of respondents were male (56.9%), aged 19 to 39 years (67.3%). Respondents described 698 cases of SGBV (R 328, P 370), comprising 1110 acts of multi-types of violence. Respondents from Malta (160) and Belgium (143) reported the highest number of SGBV cases. The main reported causes were frustration and stress (R 23.6%, P 37.6%, *p* 0.008) and differences related with cultural background (R 19.3%, P 20.3%, *p* 0.884). Respondents assumed that these acts of violence could be prevented by SGBV prevention interventions (R 31.5%, P 24.7%, *p* 0.293); improving living conditions (R 21.7%, P 15.3%, *p* 0.232); and promoting communication (R 16.1%, P 28.2%, *p* 0.042). The majority of R were not aware of existing preventable measures in the asylum facility or host country. While the majority of P were aware of existing preventable measures in the asylum facility or country. Proposed SGBV prevention strategies in EARF included SGBV sensitization and awareness, improving living conditions and improving communication between R and P.

**Conclusion:**

In the EARF context, SGBV is characterized by multi-types of violence acts, yet R and P believe that prevention is possible. Our results call for urgent integrative prevention strategies that are in line with country-level and international regulations.

## Background

Sexual and Gender-based Violence (SGBV) is a widespread public health issue and a violation of human rights [1, 2] rooted in gender and power inequities [3]. Moreover, SGBV induces a wide range of health sequelae that range from physical consequences to emotional, psychological, sexual and/or reproductive health impacts [4–6]. Yet, as a result of victimization, social stigma, fear or discrimination may impede their familial and community well-being and active participation in society [4, 6, 7].

Migrants, refugees and asylum-seekers (AS) are considered a vulnerable group for sexual and reproductive diseases, including SGBV [6, 8–12]. In the context of (forced) migration, SGBV is defined as any act of violence inducing physical, psychological or sexual suffering, threats, coercion or deprivation of freedom on the basis of a person’ sex or gender [4]. SGBV comprises five dimensions of violence – physical, psychological, sexual, socio-economic and harmful cultural practices [4]. The Socio-Ecological model is used to comprehend the complexity of SGBV problematic [4, 13, 14], the implication being that there is no single cause for victimization and/or perpetration. Therefore, SGBV is considered an outcome of multiple factors that can be grouped into four interacting levels – individual, relationship, community and society [15, 16].

The incidence of SGBV towards refugees, AS and migrants is high [3, 8, 17–21]. A systematic review on violence and health concerns among AS, in high-income host countries found a 35.7% prevalence of sexual harassment in detention centres perpetrated by detention officers; a sexual violence prevalence of 44.2% reported by AS in the context of medical consultation; and four studies reporting sexual torture methods among torture victims [22]. A study conducted in Belgium and the Netherlands on the nature of SGBV that refugees, AS and undocumented migrants had experienced since arrival in Europe reported a high incidence of multi-types of violence (332 experiences reported) including sexual harassment, gang or multiple rapes and sexual exploitation [11]. Considering the European context of asylum reception facilities, the risk of SGBV is constant and the incidence high [8, 17, 19]. Indeed, research shows that SGBV vulnerability, especially of women and girls, can increase due to inadequate living conditions, overcrowding at reception facilities, lack of gender-sensitive in asylum procedures and at reception facilities [17, 19, 23]. In Europe, a country-wise distribution of SGBV incidence in refugees, AS and migrants is still inexistent [4, 8]. However, data exists on sexual aggression among young adults, in Europe. In this study, the highest one-year prevalence rate of female victimization or since the age of consent was found in the Netherland, Germany, Sweden, Spain, Finland and Greece, in opposition with Belgium, Portugal, Malta and Hungary. Even though this study assesses the available evidence of SGBV and compares studies with different methodology, sample composition and sexual aggression definition, it gives a clear picture of the dimension of SGBV problematic in Europe [5].

Although evidence exists that SGBV can be prevented [15, 24, 25] effective interventions are still not clearly identified [26]. A systematic review of evaluations of primary prevention strategies for sexual violence has concluded that only three programs have proved to have a positive impact on prevention [27]. Indeed, it is clear that prevention of SGBV should be rooted in a public health approach [24, 28–30]. Already in 2003, the European Council Directive 2003/9/CE stated that victims of rape and sexual violence should receive specific treatment, and reception facilities should be prepared to address them [31, 32]. The recast of 2013 (European Council Directive 2013/33/EU) laying down standards for the reception of applicants for international protection stated that reception and asylum facilities should implement “appropriate measures that prevent gender-based violence including sexual assault and harassment” [33]. Recently, the Center for Disease Control has launched a technical package on preventive strategies addressed to communities and states to reduce the incidence and consequences of sexual violence [15]. Even though achievements have been made, a lack on research addressing the specific context of asylum reception facilities exists [8, 34].

Acknowledging that SGBV is a preventable public health problem we aim to explore reported cases of SGBV described by residents – refugees, AS and migrants – and professionals, living and working in European Asylum Reception Facilities (EARF), causes and preventable measures. The reported cases take into account the violence that was witnessed and/or experienced, violence that was committed among residents and violence committed by professionals towards residents or vice versa. Furthermore, we intended to analyze potentially preventable measures of SGBV described by the same population. In this sense, we are committed to contributing to the development of evidence-based SGBV prevention measures, adopting a public health approach.

## Methods

### Conceptual model

Our conceptual framework was founded on a public health approach to violence [16] and the Socio-ecological Model [14] to address SGBV. From a public health perspective, primary prevention is considered the most effective way to prevent violence having a population-level effect [30]. In this sense, to understand and prevent violence four fundamental steps should be taken into account: (1) defining the problem, (2) identifying the factors that increase the risk for violence, (3) developing and testing prevention strategies, and (4) disseminating and implementing broadly [16]. The Socio-ecological model uses a multi-factorial system to understand SGBV causes, consequences and subsequent preventable measures [4, 13]. Indeed, this model recognizes that “(…) events in higher-order social ecosystems should influence human development through their impact on events in lower-order social ecosystems” [35]. It incorporates four-level factors –individual, relationship, community and society – to understand, mitigate and prevent violence [15, 16, 36]. In this sense, we consider that primary prevention measures should focus on the contributing factors for violence at four dynamic levels – individual, relational, community and society. Through the analysis of reported cases of SGBV, we will apply a four-level perspective in order to enhance primary prevention strategies for SGBV, in this specific context – EARF.

### Senperforto project

A cross-sectional study was developed using data from a knowledge, attitude and practice (KAP) questionnaire from the Senperforto Project. The objective of the Senperforto project was to develop a gender-balanced European Frame of Reference adapted for professionals and residents from EARF [37]. Professionals were defined as service or health care providers working in the EARF, for or with the residents, being refugees, AS and/or unaccompanied minors. A KAP questionnaire among residents and professionals of the asylum reception sector in 8 partner countries was conducted. The questionnaire included three areas of research: the first regarding knowledge on types of SGBV, on experiences and on existent preventable measures; the second regarding their attitudes towards SGBV within EARF; and finally, the third comprising the existence of violence prevention and response measures, and needs to enhance it and to mitigate the vulnerability.

Stakeholders from the eight European countries were included in community advisory boards (CAB) from the eight participating countries. These CAB consisted of AS and refugees, asylum reception professionals, policymakers, intermediary organizations, civil society and researchers engaged in the asylum and reception sector. The CAB were intermediaries who have a critical and distinctive impact on the process, the result and participated in every decisive phase of the project.

A detailed description of Senperforto Project is available in the published paper *Sexual and Gender-based violence in the European asylum and reception sector: a perpetuum mobile* [8].

### Population, sampling and data collection

Our study sample (*n* = 600) comprises residents (refugees, AS and migrants) and professionals (service and health care providers) from EARF in eight European countries: Belgium, Ireland, Malta, Greece, Hungary, Portugal, Netherland and Spain. Sampling for the Senperforto study took into consideration residents and professionals, living and working in EARF as our main beneficiaries. Further, sampling included the most numerous groups of AS per partner country, covered all different types of reception facilities within a partner country, gender-balanced and geographical distribution; and random sampling within the pool of people matching these criteria. A geographical distribution of all official and unofficial reception facilities over the 8 countries of research was done. Random sampling was applied in six of the eight countries - Belgium, Ireland, Malta, Greece, Hungary and Portugal. Then we obtained the permission of the sampled facilities and we applied the inclusion criteria. Finally, the respondents were randomly sampled from their list of residents and professionals. In the Netherlands and Spain, a convenience sampling was adopted due to political constrains. A complete description of the inclusion criteria and sampling methods is available in the previously mentioned published article [8]. Data was collected through semi-structured interviews based on a KAP questionnaire and implemented by trained community researchers (CR). The data on country of research, living conditions of residents within EARF and awareness of respondents regarding the existence of preventable measures was collected through closed questions. For the data on SGBV reported cases, causes and preventable measures we used open questions.

### Data analysis

At first data cleaning, we excluded all the Spanish interviews for the open questions part regarding violence experiences, attitudes and prevention and response measures. The notes and transcriptions of the CR were scarce and inconsistent and we doubted whether the validity of the data could be guaranteed [8]. This brought the total of analyzed interviews down to 562 interviews: 375 with residents and 187 with professionals. For descriptions of violence exposure, causes and preventable measures, we firstly applied a framework analysis technique to categorize types of violence, perpetrators, victims and relations. Data was entered into IBM® SPSS software. For this process three researchers were involved, they previously have agreed on a set of categories that were then included in the database. Quantitative data from closed questions was entered directly in IBM® SPSS software database. Data analysis comprises statistical tests – *Chi-square Test* and *Fisher’s exact test –*, to analyze if a significant statistical association exists at the 5% significance level. Fisher’s exact test was specifically used for tables with expected cell frequencies less than 5.

The Senperforto study protocol applied the WHO and UNHCR ethical and safety guidelines in researching violence, complied with the local ethical requirements and received ethical approval from Ghent University Hospital Ethical Committee [B67020096667].

## Results

Respondents included 562 persons: 375 (66.7%) residents and 187 (33.3%) professionals. The majority of respondents were male (56.9%), aged from 19 to 39 years (67.3%). Residents mostly had asylum seeker status (60.3%), while professionals had national citizenship (87.2%). The majority of residents lived in an open reception centre (74.0%), more specifically in a room (45.3%) or house/apartment (41.6%), with a common area (89.3%) and a place to sleep (97.9%) of 2-4 m^2^ (43.0%). Residents shared this place with 1 to 2 adults (40.5%), with whom they had no relation (52.8%) while the majority shared the space with their own children (65.1%). Further details of residents’ living conditions in EARF are presented in Table 1.

**Table 1:** Living conditions of Residents at European asylum reception facilities

	Residents		
Living conditions at EARF	Female *N* (%)	Male *N* (%)	Total *N* (%)
Type of accommodation			
Room	62 (45.3)	84 (35.4)	146 (39.0)
House or apartment	57 (41.6)	85 (35.9)	142 (38.0)
Shelter	5 (3.6)	31 (13.1)	36 (9.6)
Studio or container	11 (8.1)	20 (8.4)	31 (8.3)
Tent or homeless	0 (0.0)	10 (4.2)	10 (2.6)
Other	2 (1.5)	7 (3.0)	9 (2.4)
Missing	–	–	1
Place to sleep – size m^2^			
2–4	58 (43.0)	84 (36.5)	142 (38.9)
6–8	43 (31.9)	56 (24.3)	99 (27.1)
10–12	24 (17.8)	40 (17.4)	64 (17.5)
14–20	6 (4.4)	44 (19.1)	50 (13.7)
more 22	4 (3.0)	6 (2.6)	10 (2.7)
Missing	–	–	–
With how many adults do you share this space?			
0	12 (14.3)	2 (1.2)	14 (5.5)
1 to 2	34 (40.5)	61 (35.9)	95 (37.4)
3 to 5	24 (28.6)	50 (29.4)	74 (29.1)
> 6	14 (16.7)	57 (33.5)	71 (27.9)
Missing	–	–	121
What is their relationship to you?			
Not related	38 (52.8)	78 (48.4)	116 (49.8)
(Co-) resident(s)	19 (26.4)	43 (26.7)	62 (26.6)
Partner Family or Friend(s)	15 (20.8)	40 (24.8)	55 (23.6)
Missing	–	–	142
With how many children do you share this space?			
0	19 (24.1)	63 (50.4)	82 (40.2)
1 to 2	44 (55.7)	41 (32.8)	85 (41.7)
3 to 5	12 (15.2)	10 (8.0)	22 (10.8)
> 6	4 (5.1)	11 (8.8)	15 (7.3)
Missing	–	–	171
What is their relationship to you?			
Own Children	41 (65.1)	9 (15.5)	50 (41.3)
No-Relationship	17 (27.0)	38 (65.5)	55 (45.5)
Family or friends	2 (3.2)	7 (12.1)	9 (7.5)
(Co-) residents	3 (4.8)	4 (6.9)	7 (5.8)
Missing	–	–	254

### Reported cases of SGBV

Respondents were asked to describe cases of SGBV that they recalled in the year prior to the interview [Table 2]. In total 698 cases were described: residents reported 328 cases and professionals reported 370. Regarding the distribution of reported cases per country, residents from Belgium (67 cases) and Ireland (67 cases) described the highest number of cases. For professionals, respondents from Malta have reported the highest number of SGBV cases (99 cases) [Table 3].

**Table 2:** SGBV Cases reported by residents and professionals and gender

	Case report *N* (%)			No case report *N* (%)			Case report *N* (%)			No case report *N* (%)		
	Residents						Professionals					
	Female	Male	Total	Female	Male	Total	Female	Male	Total	Female	Male	Total
First CASE^a^	71 (37,8)	117 (62,2)	188 (50.1)	66 (35,3)	121 (64,7)	187 (49.9)	73 (52,5)	66 (47,5)	140 (74.9)^d^	32 (68,1)	15 (31,9)	47 (25.1)
Second CASE^b^	36 (37,5)	60 (62,5)	96 (25.6)	98 (35,8)	176 (64,2)	274 (73.1)	66 (56,9)	50 (43,1)	116 (62.4)	39 (55,7)	31 (44,3)	70 (37,6)
Third CASE^c^	14 (38,9)	22 (61,1)	36 (9.6)	123 (36,3)	216 63,7)	339 (90.4)	49 (58,3)	35 (41,7)	84 (44.9)	56 (55,4)	45 (44,6)	101 (54,6)
Fourth CASE^a^	4 (50,0)	4 (50,0)	8 (2.1)	133 (36,2)	243 (63,8)	367 (97.9)	19 (63,3)	11 (36,7)	30 (16.0)	86 (55,1)	70 (44,9)	156 (83,9)
Total cases reported	328						370					

^a^No missing values, for both groups

^b^For the description of the second case, five missing answers for residents

^c^For the description of the second case, one missing answer for professionals

^d^One professional did not describe gender

**Table 3:** Number of reported cases of SGBV by residents and professionals, per country

	Residents		Professionals		Total	
Country	*N*	Reported cases	*N*	Reported cases	*N*	Total cases
Belgium	61	67	32	76	93	143
Greece	36	27	30	60	66	87
Hungary	68	35	21	66	89	101
Ireland	63	67	32	55	95	122
Malta	61	61	30	99	91	160
The Netherlands	33	61	5	10	38	71
Portugal	53	10	37	4	90	14
TOTAL	375	328	187	370	562	698

Considering the description of acts of violence per SGBV reported case [Table 4], it was as follow: for residents, 50.1% reported one single case of SGBV, 25.6% reported two cases, 9.6% reported three cases and only 2.1% of residents reported a fourth case of SGBV. Residents’ SGBV reporting included 207 (40.6%) acts of physical violence, 192 (37.6%) acts of psychological violence, 84 (16.5%) acts of socio-economic violence and 27 (5.3%) acts of sexual violence. For professionals, we found that more than half of respondents reported a first and second case of SGBV when asked, 74.9 and 62.0%, respectively (see Table 2). Professionals’ SGBV reporting included 259 (43.2%) acts of physical violence, 260 (43.3%) acts of psychological violence, 43 (7.2%) acts of socio-economic violence and 38 (6.3%) acts of sexual violence. Neither of the two groups described acts of harmful cultural practices. In sum, from the 698 cases described, 1110 acts of multi-types of violence were included.

**Table 4:** Number of acts of violence reported by residents and professionals

Total Acts Violence	Residents			Professionals			Total Acts
	Female	Male	Acts *N* = 510 (%)	Female	Male	Acts *N* = 600 (%)	*N* = 1110 (%)
Physical Violence	207 (40.6)			259 (43.2)			466 (42.0)
Singular non life-threathening	53 (25.6)	73 (35.3)	126 (24.7)	72 (27.8)	58 (22.5)	131^a^ (21.8)	257 (23.2)
Multiple non life- threathening	11 (5.3)	18 (8.7)	29 (5.7)	24 (9.3)	16 (6.2)	40 (6.7)	69 (6.2)
Singular life- threathening	13 (6.3)	23 (11.1)	36 (7.1)	34 (13.1)	25 (9.7)	59 (9.8)	95 (8.6)
Multiple life- threathening	2 (1.0)	11 (5.3)	13 (2.5)	18 (6.9)	10 (3.9)	28 (4.7)	41 (3.7)
Killing	1 (0.5)	2 (1.0)	3 (0.6)	1 (0.4)	0 (0.0)	1 (0.2)	4 (0.4)
Psychological Violence	192 (37.6)			260 (43.3)			452 (40.7)
Verbal violence	34 (17.7)	43 (22.4)	77 (15.1)	46 (17.7)	43 (16.5)	89 (14.8)	166 (15.0)
Humiliation	23 (12.0)	52 (27.1)	75 (14.7)	23 (8.8)	22 (8.5)	45 (7.5)	120 (10.8)
Threatening	9 (4.7)	20 (10.4)	29 (5.7)	46 (17.7)	39 (15.0)	85 (14.2)	114 (10.3)
Confinement	1 (0.5)	3 (1.6)	4 (0.8)	3 (1.2)	3 (1.2)	6 (1.0)	10 (0.9)
Relational violence	6 (3.1)	1 (0.5)	7 (1.4)	25 (9.6)	9 (3.5)	35^a^ (5.8)	42 (3.8)
Sexual Violence	27 (5.3)			38 (6.3)			65 (5.9)
Sexual harrasment	6 (22.2)	6 (22.2)	12 (2.4)	11 (28.9)	10 (26.3)	21 (3.5)	33 (3.0)
Sexual abuse	3 (11.1)	3 (11.1)	6 (1.2)	4 (10.5)	2 (5.3)	6 (1.0)	12 (1.1)
Attempt to rape	1 (3.7)	0 (0.0)	1 (0.2)	0 (0.0)	0 (0.0)	0 (0.0)	1 (0.1)
Rape	0 (0.0)	3 (11.1)	3 (0.6)	2 (5.3)	1 (2.6)	4^a^ (0.7)	7 (0.6)
Sexual exploitation	2 (7.4)	3 (11.1)	5 (1.0)	5 (13.2)	2 (5.3)	7 (1.2)	12 (1.1)
Harmfull Cultural Practices	0 (0.0)	0 (0.0)	0 (0.0)	0 (0.0)	0 (0.0)	0 (0.0)	0 (0.0)
Socio-economic Violence	84 (16.5)			43 (7.2)			127 (11.4)
Discrimination	9 (10.7)	21 (25.0)	30 (5.9)	7 (16.3)	7 (16.3)	14 (2.3)	44 (4.0)
Refusal of assistance	18 (21.4)	27 (32.1)	45 (8.6)	9 (20.9)	16 (37.2)	25 (4.2)	68 (6.1)
Social exclusion	4 (4.8)	3 (3.6)	7 (1.4)	0 (0.0)	2 (4.7)	2 (0.3)	9 (0.8)
Refusal of legal protection	0 (0.0)	2 (2.4)	2 (0.4)	1 (2.3)	1 (2.3)	2 (0.3)	6 (0.5)

^a^One missing value

### Causes of reported SGBV cases

In order to understand the causes that trigger SGBV in EARF, respondents were asked about their assumption of the main causes of reported violence. From our total sample only 161 (42.9%) residents and 133 professionals (71.1%) answered the question (n total = 294). Table 5 presents the presumed causes of SGBV as framed by the respondents. Residents reported as main causes: frustration and stress (23.6%), different cultural, ethnic backgrounds and practices (19.3%), asylum procedures (13.7%), communication problems (9.9%) and bad accommodation (8.7%). Further, male residents were more likely to report that “staff competence” was a cause for violence (*p-value* 0.012). Causes of violence mostly mentioned by professionals were frustration and stress (37.6%), different cultural and ethnic backgrounds and practices (20.3%), communication problems (11.3%). For this group no significant statistical associations in gender were found (*p-value* 0.501, Fisher test).

**Table 5:** Causes of reported cases of SGBV

	Residents			Professionals			TOTAL		
Causes of reported SGBV	Female *N* (%)	Male *N* (%)	*p*	Female *N* (%)	Male *N* (%)	*p*	Resid *N* (%)	Profs *N* (%)	*p*
Coping (frustration & stress management)	14 (8.7)	24 (14.9)	0.709	30 (22.6)	20 (15.0)	0.369	38 (23.6)	50 (37.6)	**0.008**
Different cultural/ethnic backgrounds & practices	15 (9.3)	16 (9.9)	0.310	14 (10.5)	13 (9.8)	0.831	31 (19.3)	27 (20.3)	0.884
Communication problem	8 (5.0)	8 (5.0)	0.426	8 (6.0)	7 (5.3)	1.000	16 (9.9)	15 (11.3)	0.849
Asylum procedure related	5 (3.1)	17 (10.6)	0.102	1 (0.8)	4 (3.0)	0.179	22 (13.7)	5 (3.8)	**0.004**
Bad accommodation	8 (5.0)	6 (3.7)	0.252	5 (3.8)	1 (0.8)	0.218	14 (8.7)	6 (4.5)	0.170
Multifactorial	1 (0.6)	8 (5.0)	0.088	4 (3.0)	5 (3.8)	0.732	9 (5.6)	9 (6.8)	0.808
Competence staff	0 (0.0)	9 (5.6)	**0.012**	7 (5.3)	5 (3.8)	0.774	9 (5.6)	12 (9.0)	0.363
Alcohol Abuse	1 (0.6)	1 (0.6)	1.000	2 (1.5)	5 (3.8)	0.246	2 (1.2)	7 (5.3)	0.084
Food	3 (1.9)	4 (2.5)	1.000	0 (0.0)	0 (0.0)	–	7 (4.3)	0 (0.0)	**0.017**
I don’t know	7 (4.3)	3 (1.9)	0.053	0 (0.0)	0 (0.0)	–	10 (6.2)	0 (0.0)	**0.002**
Others	2 (1.2)	1 (0.6)	0.563	1 (0.8)	1 (0.8)	1.000	3 (1.9)	2 (1.5)	1.000
Missing	–	–	–	–	–	–	214	54	–
TOTAL	64 (39.8)	97 (60.2)	**0.013**	72 (54.1)	61 (45.9)	0.501	375 (66.7)	187 (33.3)	**0.000**

Bolded significant *p*-value < 0.05

### Preventable measures of SGBV

According to respondents, 73.6% of reported cases of SGBV could be prevented (26.4% answered it could not be prevented; 225 persons did not answer the question). From the respondents that believed that this violence could be prevented the majority were residents (66.9%, *p-value* 0.000). Table 6 describes potentially preventable measures suggested for these cases of SGBV. Statistical tests conducted to explore possible associations regarding preventable measures described by the groups of residents and professionals indicated no differences (*p-value* 0.226, Fisher test). Regarding statistical associations by gender, for both groups no differences were found *(p-value* 0.940, *p-value* 0.944, respectively).

**Table 6:** Potential preventable measures to reported cases of SGBV, by residents and professionals

	Residents			Professionals			Total		
	Female *N* (%)	Male *N* (%)	*p*	Female *N* (%)	Male *N* (%)	*p*	Resid *N* (%)	Profs *N* (%)	*p*
Improved SGBV /Intervention measures	16 (28.6)	29 (33.3)	1.000	13 (26.0)	8 (22.9)	0.803	45 (31.5)	21 (24.7)	0.293
Improved accomodation & living conditions	12 (21.4)	19 (21.8)	1.000	8 (16.0)	5 (14.3)	1.000	31 (21.7)	13 (15.3)	0.232
Improved competence staff/ communication with residents	8 (14.3)	15 (17.2)	0.653	12 (24.0)	12 (34.3)	0.335	23 (16.1)	24 (28.2)	**0.042**
Coping (frustration & stress management)	6 (10.7)	9 (10.3)	1.000	6 (12.0)	4 (11.4)	1.000	15 (10.5)	10 (11.8)	0.829
Improved asylum procedure	7 (12.5)	5 (5.7)	0.217	3 (6.0)	1 (2.9)	0.640	12 (8.4)	4 (4.7)	0.606
Intercultural respect & tolerance	4 (7.1)	5 (5.7)	0.737	3 (6.0)	3 (8.6)	0.687	9 (6.3)	6 (7.1)	1.000
Improved communication between residents	2 (3.6)	3 (3.4)	1.000	1 (2.0)	1 (2.9)	1.000	5 (3.5)	2 (2.4)	0.714
Other	1 (1.8)	1 (1.1)	1.000	4 (8.0)	1 (2.9)	0.644	2 (1.4)	5 (5.9)	0.106
I don’t know	0 (0.0)	1 (1.1)	1.000	0 (0.0)	0 (0.0)	–	1 (0.7)	0 (0.0)	1.000
Missing values	–	–	–	–	–	–	232	102	–
TOTAL answers	56 (39.2)	87 (60.8)	0.940	50 (58.8)	35 (41.2)	0.944	375 (66.7)	187 (33.3)	0.226

Bolded significant *p*-value < 0.05

In addition, our respondents were asked about the existence of preventable measures in the asylum reception facility where they lived or worked, and also in their hosting country. Regarding existing preventable measures at asylum reception facilities, the majority of residents (58.3%) were not aware of existing preventive measures. For professionals 65.0% were aware of existing preventive measures in the asylum facility. Considering existing preventable measures at country-level, the majority of residents were not aware of existing preventable measures (72.4%). While 68.8% of professionals were aware of preventable measures in their country [Table 7]. Furthermore, residents who reported the existence of preventable measures also reported that the measures were effective (68.9%). The same result was found for professionals (76.3%). In the context of EARF, we found significant statistic associations between being a esident or professional and having the knowledge on existent preventive measures in the hosting country and in the reception facilities (*p-values*: 0.000, and 0.000, respectively).

**Table 7:** Existence of preventable measures at country level and reception asylum facility

		Residents					Professionals					Total				
		Female		Male			Female		Male			Resid N 375 (66.7)		Profs N 187 (33.3)		
		N	%	N	%	*p*	N	%	N	%	*p*	N	%	N	%	*p*
Existing preventive measures in the hosting country?	Yes	41	31.3	59	25.5		61	58.7	55	68.8		100	27.6	117	63.2	
	No	90	68.7	172	74.5		43	41.3	25	31.3		262	72.4	68	36.8	
Total		131	36.2	231	63.8	0.271	104	56.5	80	43.5	0.169	362	66.2	185	33.8	**0.000**
Missing		–	–	–	–		–	–	–	–		13	–	2	–	
Any preventive measures in the reception/asylum facility?	Yes	54	43.2	91	40.8		66	62.9	53	68.8		145	41.7	119	65.0	
	No	71	56.8	132	59.2		39	37.1	24	31.2		203	58.3	63	35.0	
Total		125	35.9	223	64.1	0.734	105	57.7	77	42.3	0.434	348	65.5	183	34.5	**0.000**
Missing		–	–	–	–		–	–	–	–		27		5		

Bolded significant *p*-value > 0.005

Respondents were asked about possible preventive strategies that could work in a preventive way at EARF. Table 8 presents the main answers for both groups (Rate answers: residents 44.5%, professionals 54%). Statistical differences between what residents and professionals described as possible preventable measures were found (*p-value* 0.001). No significant associations were found in gender for residents and professionals.

**Table 8:** Possible preventable measures to SGBV described by respondents

	Residents			Professionals			Total		
	Female N 57 (%)	Male N 110(%)	*p*	Female n 54(%)	Male N 47 (%)	*p*	Resid	Profs	*p*
More SGBV sensitization & awareness	9 (15.8)	17 (15.5)	1.000	15 (27.8)	13 (27.7)	1.000	26 (15.6)	28 (27.7)	**0.019**
Improve accommodation & living conditions	14 (24.6)	10 (9.1)	**0.010**	9 (16.7)	9 (19.1)	0.798	24 (14.4)	18 (17.8)	0.490
Improve communication between staff and residents	9 (15.8)	19 (17.3)	0.832	8 (14.8)	4 (8.5)	0.373	28 (16.8)	12 (11.9)	0.295
Improve prevention measures	4 (7.0)	14 (12.7)	0.304	8 (14.8)	8 (17.0)	0.791	18 (10.8)	16 (15.8)	0.258
More adequate interventions & sanctions after SGBV	6 (10.5)	6 (5.5)	0.343	4 (7.4)	5 (10.6)	0.730	12 (7.2)	9 (8.9)	0.643
More security & surveillance	2 (3.5)	8 (7.3)	0.497	4 (7.4)	3 (6.4)	1.000	10 (6.0)	7 (6.9)	0.799
Cohesion and empowerment of residents	0 (0.0)	9 (8.2)	**0.029**	1 (1.9)	2 (4.3)	0.596	9 (5.4)	3 (3.0)	0.544
Nothing	1 (1.8)	6 (5.5)	0.424	4 (7.4)	3 (6.4)	1.000	7 (4.2)	7 (6.9)	0.398
Not specified	0 (0.0)	5 (4.5)	0.167	0 (0.0)	0 (0.0)	–	5 (3.0)	0 (0.0)	0.160
I don’t know	12 (21.1)	16 (14.5)	0.382	1 (1.9)	0 (0.0)	1.000	28 (16.8)	1 (1.0)	**0.000**
*Missing*							208	86	
Total	57 (34.1)	110 (65.9)	0.024	54 (53.5)	47 (46.5)	0.979	375 (66.7)	187 (33.3)	**0.001**

Bolded significant *p*-value < 0.05

## Discussion

### Reported cases of SGBV

Our research explored reported cases of SGBV in the year prior to the interview, its assumed causes and preventable measures described by residents and professionals in the context of EARF. The results suggest a high incidence of SGBV reported by residents and professionals. Countries reporting the highest incidence of SGBV cases were Malta, Belgium, Ireland and Hungary. Greece, The Netherlands and Portugal reported less cases. A high number of combined types of SGBV was described, which is consistent with previous research on refugees, AS and migrants [11, 12, 15, 19]. For both groups, physical and psychological violence were the most prevalent types of reported violence, followed by socio-economic and sexual violence. Harmful cultural practices were not described in the reported cases. This finding is aligned with the difficult to reach, sensitive issue and social taboo that these practices represent [38]. Furthermore, residents have described fewer cases than professionals, which could be related to fear of stigmatization or expulsion of the proper community, to fear of deportation by host country officials or to barriers in communication [3, 10, 23].

### Causes of reported SGBV cases

Stating the need for identifying and understanding SGBV causes and contributing factors to develop evidence-based preventive strategies [3, 15], we highlight the main causes reported by residents and professionals. Both groups reported coping skills, as frustration and stress management, and differences related to cultural background, as main causes. In addition, they refer to communication problems as a possible cause for SGBV reported cases. Both groups emphasized the need for improving communication between staff and residents as a preventive measure to mitigate SGBV. Our results are aligned with previous research on migrants’ health, supporting policies to enhance communication as a positive outcome of health and social care for refugees and AS [10]. The need for improving communication between AS and medical systems is acknowledged as a step to overcome barriers to accessing health services, cultural issues, structural and bureaucratic problems [10].

Furthermore, residents identified the asylum procedure as a cause for the described violence. Evidence exists on identifying restricted legal status as increasing vulnerability of refugees to violence [8, 11, 23]. Victimization before and during the (forced) migration journey has been documented. The lack of laws regulating violence perpetration and the lack of support for survivors have left women more vulnerable to victimization [9]. Recent findings in asylum reception centres in Germany, state that the current time-consuming within the asylum procedure, leads to overcrowding and inadequate living conditions, which increases female residents’ vulnerability to violence [17]. We believe that the asylum procedure should be gender-sensitive, protecting all genders and promoting a SGBV free environment. Our results suggest that asylum procedure should be considered a determinant for SGBV vulnerability. Moreover, a gender-sensitive and equitable asylum procedure is an urgent need. The in-vigor Directive 2013/32/EU [39] recognizes the need for a gender-sensitive asylum procedure, ensuring that staff are also aware of gender-specific vulnerabilities. Acknowledging that achievements have been made, recent evidence shows the need for moving from theory to the reality of asylum reception centres [17].

Interestingly, alcohol abuse was only described as a potential cause for violent behavior in 8/562 cases. Previous research has linked alcohol abuse with sexual harassment, aggression or rape victimization [11, 40]. In our results, an underestimated bias should be considered. It is still important to mitigate the odds of alcohol-related aggression, not only on this specific context but also in general.

Our results demonstrate contributing factors to SGBV at different levels – individual, relational, community and societal level, aligning with the Socio-ecological model [4, 14, 41] and reinforces the concept of multifactorial causes of SGBV and the inherent complexity of addressing it. Analyzing it from a dynamic and interactive perspective, frustration and stress as a cause of violent acts can be related to bad accommodation. Living conditions previously described by residents are poor, and sharing accommodation with adults and children, male and female, with no relationship, should be considered a stressor and trigger to SGBV victimization and/or perpetration. Recent research identifies bad accommodation as an increasing factor of refugee women’s vulnerability to SGBV [17, 23]. Further, current living conditions for refugees - women and girls – in Greek Islands are described as being far from standards that mitigate SGBV victimization [19]. In our results, no gender association was found, we speculate that inadequate living conditions can be a trigger to SGBV victimization for both women and men.

### Preventable measures of SGBV

In the context of EARF, professionals and residents described potentially preventable measures for reported cases of SGBV as follows: to improve SGBV prevention and intervention measures; improve accommodation and living conditions; improve staff skills and communication with residents; improve coping strategies (frustration & stress management); improved asylum procedure; improve intercultural awareness; improve communication skills between residents. Furthermore, the groups described similar measures that could be implemented in the context of EARF. Our results are aligned with WHO preventive strategies to reduce multi-types of violence [16] and consistent with the WHO report findings, suggesting that even though countries are investing in violence prevention, the implementation of the programs does not reach the level of implementation necessary to combat the issue [16]. In our study, specific causes and preventable measures were described. However, a high incidence of cases are still reported. Respondents reported the need of awareness and intervention on SGBV and of improving preventive measures, in this sense, we highlight the need for more training on SGBV in this specific context. Our results suggest that the majority of residents are not aware of existent preventable measures at asylum and host country level. Whilst the opposite was found for professionals, they still reported the need of more SGBV education as the main preventive measure for SGBV. We believe that this is an urgent call for action urges in terms of training. We stress the need for well-defined preventive measures that can combat the problem [16]. Specific interventions should be considered, such as implementing systematic training on awareness, conceptualization, vulnerable groups and prevention of SGBV, including workshops on coping strategies to stress and frustration; improving the asylum procedure; improving basic living conditions and promoting an environment where residents and professionals can openly and respectfully communicate. Being mindful that all these interventions should ensure the respect for cultural beliefs. Yet, there is a need to go beyond the definition of preventive measures, and guarantee the implementation of interventions in the field. Moreover, a systematic evaluation of preventive measures in EARF context should take place, to ensure effectiveness.

For the group of residents, we found that gender was associated with the need of improving accommodation and living conditions and, the need for cohesion and empowerment of residents. The majority of female residents have described the need for improving accommodation and living conditions as a preventive measure in the context of asylum facilities. Which is consistent with a recent study emphasizing SGBV vulnerability of female refugees due to inadequate living conditions [17]. Yet, only male residents have described the need for cohesion and empowerment of residents. Even though associations were found, and if we take into account the assumed causes, we can assume that both genders are vulnerable to SGBV. In this sense, we consider of most importance to identify and implement preventive measures that will reinforce gender equity and reduce power imbalance, while addressing all gender needs.

Considering the European Council Directive 2003/9/CE – laying down minimum standards for the reception of asylum seekers [31] prevailing by the time the Senperforto project was conducted, we believe that a gap on clear preventive strategies of SGBV in the context of EARF existed. Supporting that is the fact that both groups have described SGBV sensitization and improvement of prevention measures as a preventable measure that could reduce SGBV victimization. According to our study, respondents have identified potential strategies that could tackle down the problematic of SGBV. We consider of most importance to promote effective communication between staff and residents to achieve a culturally sensitive environment and reduce cultural/ethnic/religious barriers. Besides, a competent, committed and connected staff is a prerequisite to effective SGBV preventive programs [27]. Accommodation and living conditions are basic factors to mitigate SGBV in this context, taking into account that a high number of SGBV cases is reported in asylum settings [8].

The European Directive 2013/33/EU laying down standards for the reception of applicants for international protection (recast) in force, replaced the Council Directive 2003/9/CE. A more in-depth Directive is in vigor, recommending that EU Member States should implement specific measures addressing SGBV, including sexual assault and harassment, and that adequate medical and psychological care for vulnerable groups should be guaranteed [33]. Even though a narrow definition for sexual violence is applied “a) focusing solely on female victimization, b) ignoring the most vulnerable among the vulnerable migrants (undocumented, LGBT, sex workers, …) and c) focusing predominantly on victimization in the countries (sexual violence as a weapon of war, torture, trafficking) or cultures of origin (e.g. FGM)” [32]. We believe that the different countries researched have implemented this Directive to different levels. European countries must ensure an effective implementation of minimum standards at asylum reception facilities [42]. Taking into account the living conditions of our respondents and the identification of it as potential preventive measures, we believe that specific measures should be considered a priority. Big steps have been taken in this matter, and in 2016, the European Asylum Support Office (EASO) released the “EASO guidance on reception conditions: operational standards and indicators” [43]. This guidance brings clear standards that should be present at asylum reception facilities, and corresponding indicators to evaluate the living conditions. Even though, improvements of EU Directives and EASO guidelines have been done, we believe that the recent “refugee crisis” has caused a strain in European reception facilities – increasing poor living conditions, overcrowding and lack of privacy – and a constant and high risk of SGBV victimization and/or perpetration still exists [17, 19].

Through our results, significant associations emerged for residents or professionals and the description of the specific causes for reported SGBV and potentially preventable measures. We highlight the importance of legal status, asylum-related procedures, and unequal power relations, as risk factors for SGBV victimization and/or perpetration. Furthermore, to involve professionals and residents of EARF as active stakeholders, when defining and implementing SGBV prevention measures, is primary. Also, taking into account that a high incidence of perpetrators are staff, guards or volunteers [8, 17], it is urgent to promote SGBV awareness and education, by promoting compulsory training on prevention and response policies, targeting all vulnerable groups.

Additionally, and regardless that evidence has shown that violence can be prevented [15, 16, 24], we still have residents and professionals (14/562) that believe that this is not possible. To ensure that communities at risk can be protected from being victims and/or perpetrators of SGBV it is essential to engage with professionals and residents while defining preventive measures.

Even though significant and relevant findings arise from our study we highlight the importance of acknowledging limitations of our research. First, we cannot exclude that the community researchers conducting the interviews could have had a different conceptualization of SGBV despite the standardized training. Secondly, we have no reported cases of harmful cultural practices, what can be related with the evidence that this type of violence is rarely disclosed due to sensitivity and social taboos within an asylum procedure [38]. Another relevant bias is related with the disclosure of violence. We believe that our results underestimate the reality of SGBV in EARF. Even though confidentiality was guaranteed during the interview, residents might have feared it could influence their asylum procedure. Yet, in some big asylum reception facilities where communities with honour rules were residing, it was reported to us later that residents discouraged others to participate in the study, mentioning potential stigma and/or community consequences [8]. For professionals, they assumed not to dare to speak openly, even if they had superior consent.

Finally, we believe that further research is needed addressing the specific context of asylum reception facilities, and evaluating SGBV preventive strategies [8, 15, 27]. There is an urgent need to understand the impact of SGBV preventive strategies and what works best, according to the target population and specificity of the social context. Prior research has already sustained the need for systematic evaluation research on prevention and management of all kinds of gender-based violence [25–27].

## Conclusion

Our research shows the complexity of addressing SGBV in EARF context, with a high reporting of multi-types of violence and a multi-causality of SGBV. Residents and professionals have identified potential causes that trigger SGBV. Both groups refer that the majority of SGBV reported cases could be prevented with effective preventive measures adapted to EARF.

A reflection on current preventive strategies, evidence-based on causes of SGBV, is urgently required to reduce SGBV. Considering the context of EARF, we believe there is a window of opportunity to implement integrated preventive strategies for such a complex and highly vulnerable population. We believe that residents and professionals should be considered active stakeholder to defining SGBV preventive measures. We highlight the importance of gender-sensitivity and equity in asylum procedures and adequate accommodation facilities to promote a SGBV free environment.

Taking into consideration the recent “refugee crisis” in Europe, we insist on the importance of improving preventable strategies and policies to mitigate SGBV. Even thought, SGBV incidence data is lacking, this is due to the survivors avoiding disclosure of their experience unless visible and severe health consequences arise [19]. Refugees, AS and migrants are victims of SGBV, with men and women being vulnerable [8], and the fact that this violence is committed in EARF requires an urgent call for action [17].
